# Prevalence of Foodborne Viruses in Berries Harvested in Canada

**DOI:** 10.3390/foods12040723

**Published:** 2023-02-07

**Authors:** Eva Chatonnat, Kim Manseau-Ferland, Eric Jubinville, Valérie Goulet-Beaulieu, Julie Jean

**Affiliations:** Food Science Department, Institute of Nutrition and Functional Foods, Université Laval, Quebec City, QC G1V 0A6, Canada

**Keywords:** foodborne virus, berries, food safety, HuNoV, HAV, HEV, molecular detection

## Abstract

It is known that the transmission of different foodborne viruses can occur either via discharge of contaminated water close to the production environment or via close contact with animal feces. Cranberries are intimately associated with water throughout their production cycle, and blueberries grow close to the ground which could lead to contact with wildlife. The aim of this study was to evaluate the prevalence of human norovirus (HuNoV GI and GII), hepatitis A virus (HAV) and hepatitis E virus (HEV) in two berries produced commercially in Canada. The detection of HuNoV and HAV on RTE cranberries and of HEV on wild blueberries was evaluated using the ISO method 15216-1:2017. Only 3 of 234 cranberry samples tested positive for HuNoV GI (3.6, 7.4, 5.3 genome copies/g, respectively) and all were negative for HuNoV GII and HAV. PMA pre-treatment and sequencing confirmed the absence of potential intact HuNoV GI particles on cranberries. None of the 150 blueberry samples tested positive for HEV. Overall, the prevalence of foodborne viruses in RTE cranberries and wild blueberries harvested in Canada is low, making these products relatively safe for consumers.

## 1. Introduction

Every year in Canada, more than four million individuals suffer from at least one foodborne illness. Half of these infections are due to viruses [1], intracellular infectious agents that can persist on surfaces, in water or on food for several days or years [2,3]. The most common foodborne viruses are human norovirus (HuNoV) and hepatitis A virus (HAV), with hepatitis E virus (HEV) considered as an emerging pathogen.

HuNoV is mainly transmitted via the fecal–oral route and causes gastroenteritis [4]. As many as 10 HuNoV genogroups have been recognized, but those mostly frequently implicated in foodborne illness are HuNoV GI and HuNoV GII [5]. Also spread via the fecal-oral route, HAV, which causes liver damage, can be transmitted via blood and sexual relations [6]. The transmission of these viruses via food is usually due to poor hygiene practices. Contaminated potable water or irrigation water can also lead to food contamination [7]. Canada is the world’s second largest producer of cranberries [8], a berry that is harvested after flooding the fields [9]. They are in close contact with water year-round through irrigation and frost protection, and then through flooding at harvest time [10,11]. To our knowledge, there are no data on viral contamination of RTE cranberries in Canada.

In industrialized countries, HEV causes sporadic cases of hepatitis through zoonotic transmission via HEV-3 and HEV-4 genotypes. Swine are the primary host of HEV, and virions are transmitted mainly through the consumption of raw or undercooked pork [12]. However, HEV contamination of vegetables and fruits has also been demonstrated [13,14]. Wildlife, and especially deer in Canada, are known carriers of HEV-3 and HEV-4 [15] and could contaminate crop fields or irrigation water with feces [16]. Bacterial contamination of strawberries via deer feces has been documented in the United States (USA) [17]. In Canada, blueberries and especially wild blueberries carry the risk of HEV contamination because they are grown close to the ground, in fields near forests with wildlife that could eat these fruits. However, data on HEV in berries are scarce.

Only a few studies have been conducted on the prevalence of foodborne viruses on berries in Canada [18,19]. In this study, we investigated the occurrence of viruses in ready-to-eat (RTE) cranberry and wild blueberry production. The prevalence of HuNoV GI, HuNoV GII and HAV was examined in a pilot study of RTE cranberries harvested by 44 producers in the province of Quebec, followed by a study of HEV on blueberries harvested by the five largest producers in Quebec and New Brunswick. For both studies, the virus was eluted from the samples using ISO method 15216-1:2017(E) [20]. Viral RNA was detected by performing RT-qPCR and when samples were positive, additional testing was performed with a propidium monoazide (PMA) pre-treatment followed by Sanger genome sequencing.

## 2. Materials and Methods

### 2.1. Sampling

Due to the lack of data on the prevalence of viruses in cranberries and the fact that no outbreak of viral illness has ever been reported in association with this product in Canada, our statistical hypothesis was established by considering these facts. So, we presumed a prevalence of less than 5% for HuNoV and HAV and expected about 2% in RTE cranberries grown in Quebec. For the cranberry study, 234 samples, each weighing 25.0 ± 0.3 g, were collected randomly from different lot numbers representing 44 Quebec producers during the autumn 2021 harvest (28 September to 21 November), covering a harvest area equivalent to 15% of that of Quebec [21]. The number of samples collected from each producer was proportional to the number of cultivated acres and hence to actual total production. The samples were representative of fresh, RTE cranberries and were sanitized at the distribution center, with a peracetic acid solution (50 to 80 ppm), and frozen for preservation.

To study the presence of HEV on blueberries, the choice for the number of samples was based on an expected prevalence of 2%, based off the biggest study to date mentioning this virus in association with fruits and vegetables [16]. A total of 150 samples (25.0 ± 0.3 g each) were collected from 5 producers of wild blueberries in the provinces of Quebec and New Brunswick during the 2021 harvest (16–30 August). These 5 producers were selected because their blueberry fields were near forested areas with known wildlife activity.

All cranberry and blueberry samples were stored at –30 °C until processing.

### 2.2. Sample Processing Control

In accordance with ISO method 15216-1:2017(E), Mengo virus strain vMC0 was used as a positive processing control. The viral titer was 7.41 × 10^6^ genome copies/µL for cranberries and 2.16 × 10^6^ genome copies/µL for blueberries.

### 2.3. Virus Concentration and Nucleic Acid Extraction

Cranberries and blueberries were processed frozen for analysis and treated as described in ISO method 15216-1:2017(E) [20]. Briefly, the entire sample (25.0 ± 0.3 g) was placed in a mesh filter bag (Biomérieux, Marcy-l’Étoile, France) to which 10 µL of Mengo virus suspension (1.0 × 10^5^ genome copies/µL) [22], 40 mL of tris (Sigma-Aldrich, St-Louis, MO, USA), glycine (Bio-Rad, Hercules, CA, USA), beef extract buffer (Life Technologies Corporation, Thermo Fisher Scientific, Waltham, MA, USA) and 1 mL of pectinase (from *Aspergillus aculeatus*, Sigma-Aldrich, Saint-Louis, MO, USA) were added, followed by shaking at 60 oscillations/min for 20 min at room temperature. The eluate was clarified by centrifuging at 10,000× *g* for 30 min at 4 °C, and the supernatant was mixed with 0.25 volume of 5X polyethylene glycol/NaCl solution (Fisher Chemicals, Thermo Fisher Scientific, Waltham, MA, USA), adjusted to pH 7.0 ± 0.5, shaken at 60 oscillations/min for 60 min at 4 °C and concentrated by centrifuging at 10,000× *g* for 30 min at 4 °C (the supernatant was discarded). For cranberries, the pellet was resuspended with 500 µL of phosphate-buffered saline (PBS). For blueberries, the pellet was thicker, and 1 mL of PBS was needed. The same volume of chloroform/butanol (1:1) was added and the mixture was held at room temperature for 5 min before being centrifuged at 10,000× *g* for 15 min at 4 °C, and the aqueous phase was transferred to a clean tube for RNA extraction. All processed samples were analyzed immediately or stored at 4 °C for no more than 24 h. A negative processing control with reagent only (target pathogen-free non-matrix sample) was used in all experiments and run in parallel with the samples. 

Samples were lysed with 2 mL of NucliSENS lysis buffer (Biomérieux, Marcy-l’Étoile, France) at room temperature for 10 min and then centrifuged at 1800× *g* for 2 min at 4 °C. Viral RNA was extracted on the semi-automated platform eGENE-UP (Biomérieux, Marcy-l’Étoile, France) and eluted using 100 µL of NucliSENS buffer 3. All samples and dilutions were stored at –80 °C until RT-qPCR analysis. The negative extraction control consisted of 2 mL of lysis buffer.

### 2.4. Real-Time Reverse-Transcriptase PCR

Virus (Mengo virus, HuNoV genotypes GI and GII, HAV and HEV) was detected using an adaptation of a protocol described previously [23]. Viral genome was amplified using the iTaq Universal Probe One-step (Bio-Rad, Hercules, CA, USA). The primers and probes are listed in Table 1 and were used as described in the ISO method or elsewhere in the case of HEV [24,25]. For HuNoV, HAV and HEV, a standard curve was obtained using the MiniGene pIDTSMART-AMP+ plasmid generated by IDT (Integrated DNA Technologies, Coralville, IA, USA), in triplicate at each titer, from 10^5^ genome copies/µL to 10^1^ genome copies/µL. The selected insert for HuNoV GI, GII and HAV are adapted from the ISO method (Annex G) and the sequence of the insert for HEV is GTTCCGGCGGTGGTTTCTGGGGTGACCGGGCTGATTCTCAGCCCTTCGCAATCCCCTATATTCATCCAACCAACCCCTTCGCCCCCGA, based on the original genome (NCBI Ref. Seq. NC_001434.1). For Mengo virus, the standard curve was performed in duplicate analyses of serial dilutions as described by the ISO method. RT-qPCR reactions were performed in 96-well clear PCR microplates (Applied Biosystems, Thermo Fisher Scientific, Waltham, MA, USA) and sealed with an optical adhesive film (Applied Biosystems, Thermo Fisher Scientific, Waltham, MA, USA). The ABI7500 real-time PCR thermal cycler (Applied Biosystems, Thermo Fisher Scientific, Waltham, MA, USA) was used as follows: 50 °C for 10 min, 95 °C for 3 min and 45 cycles of 95 °C for 15 s and 60 °C for 30 s. All analyses were performed using SDS Software v1.5.1 (Applied Biosystems, Thermo Fisher Scientific, Waltham, MA, USA). ROX was used as a reference dye. Each sample was analyzed simultaneously in duplicate and was diluted 1:10 to check for inhibitors using the ΔCq method. The negative control, consisting of RNase-free water (VWR, Avantor, Radnor, PA, USA), was duplicated in all cases. All standard curves had an R² over 0.985 and an efficiency of between 90% and 110%.

### 2.5. Extraction Efficiency 

Mengo virus recovery was calculated for each method to confirm the effectiveness of the elution. A recovery of higher than 1% was required for the results to be considered. The extraction efficiency was calculated as per the ISO method, using the following equation:

$$\mathrm{RNA}\;\mathrm{recovery}\;\mathrm{rate}\;(\%)=\frac{{\left[\mathrm{RNA}\right]}_{sample}}{{\left[\mathrm{RNA}\right]}_{positivecontrol}}\times 100$$

where [RNA] is the number of copies per sample.

### 2.6. Confirmation of the Presence of Virus

If one well tested positive at the first RT-qPCR, a second RT-qPCR was performed, making the analysis triplicate, again with a 1:10 dilution to check for inhibitors. If the sample tested positive again, it was considered positive. The infectious state of the virus was then tested using a pre-treatment with propidium monoazide (PMA). Finally, endpoint PCR was performed, and the product was sequenced using the Sanger method.

#### 2.6.1. Propidium Monoazide

PMA pre-treatment was conducted before the lysis step, according to an adaptation of a protocol described elsewhere [32]. Another sample (25.0 ± 0.3 g) from the same lot was weighed, and the ISO method was followed. Just before lysis, the supernatant was mixed with PMAxx^TM^ Dye (Biotium, Fremont, CA, USA) at 20 mM in H_2_O (50 µM final concentration) in a clean tube. The PMA treatment was performed according to the manufacturer’s instructions. Finally, 2 mL of lysis buffer was added, and viral RNA extraction was performed as described in Section 2.3.

#### 2.6.2. Endpoint PCR and Sequencing

Each extracted RNA sample (10 µL) was first transcribed to cDNA with 4 µL of 5X iScript reaction mix, 1 µL of reverse transcriptase and 5 µL of PCR-grade water (iScript cDNA Synthesis kit, Bio-Rad, Hercules, CA, USA) with the thermocycler (Eppendorf^®^ Mastercycler gradient, Millipore Sigma, Darmstadt, Germany) at 25 °C for 5 min, 46 °C for 20 min and 95 °C for 1 min. In total, 5 µL of the resulting cDNA was mixed with 10 µL of iQ Supemix, 1 µL each of forward and reverse primer (both 10 µM) and 3 µL of PCR water (iQ Supermix, Bio-Rad, Hercules, CA, USA) and amplified under the following conditions: 95 °C for 5 min, 45 cycles of 95 °C for 30 s, 55 °C for 30 s and 72 °C for 1 min, and when the cycles were terminated, a final elongation at 72 °C for 10 min occurred. The primers are listed in Table 2. All samples were then stored immediately at –80 °C until processing.

Prior to sequencing, the cDNA was purified by performing electrophoresis on agarose gel (2%) with tris acetate EDTA buffer 1X and SYBR^®^ Safe (0.01%, Invitrogen, Thermo Fisher Scientific, Waltham, MA, USA). The sample was mixed with 6X TriTrack DNA loading dye (Thermo Scientific, Thermo Fisher Scientific, Waltham, MA, USA) on each well according to the manufacturer’s instructions and was compared to the DNA 100 bp ladder (Thermo Scientific, Thermo Fisher Scientific, Waltham, MA, USA) after 45 min of migration at 120 V. A UV photograph was taken using the ChemiDoc^TM^ MP imaging system (Bio-Rad, Hercules, CA, USA) and processed using Quantity One^®^ software (Bio-Rad, Hercules, CA, USA).

PCR products were Sanger-sequenced on the IBIS Genomic Analysis Platform at Université Laval (Quebec City, QC, Canada) in accordance with their standard procedure [33].

**Table 2:** Specific primers used for endpoint PCR and Sanger sequencing.

Target	Sequence	References
**HuNoV GI**		
Forward primer G1SKF	CTGCCCGAATTYGTAAATGA	[34]
Reverse primer G1SKR	CCAACCCARCCATTRTACA	[34]

### 2.7. Statistical Analysis

The number of collected samples was calculated using PASS software with a binomial enumeration approach. The values used for RTE cranberries were based on the real prevalence being lower than 5% with an expected prevalence of 2%, a power of 80% and a type 1 error probability of 5%. For blueberries, the expected prevalence was 2% with a statistical test power of 95% and a type 1 error probability of 5%.

## 3. Results

### 3.1. Prevalence of HuNoV Genotypes I and II and HAV in Cranberries

RNA from HuNoV GI was detected in 3 of the 234 cranberry samples (1.28%), whereas neither HuNoV GII nor HAV were detectable (Table 3). The three positive samples were from the same region of Quebec. All positive samples had a low number of genome copies detected per gram (3.6, 7.4, 5.3, respectively). The prevalence of HuNoV and of HAV in the cranberries harvested was indeed below 5.0%, confirming our presumption prior to the pilot study. The RT-qPCR results for the positive samples are summarized in Table 4. The average percentage of Mengo virus recovery is 28.7%, and the recovery of this control virus is greater than 1% for all samples (1.2–86.9) (Table 3), which respects the validity limit of the ISO method.

### 3.2. HuNoV GI Sequence Analysis and Genotyping

The sample lots that led to positive results were analyzed again but with a PMA treatment to confirm the potential infectiousness of the detected virus [35], information that the ISO method does not provide. The positive samples were also Sanger-sequenced to identify the HuNoV GI genotype. Since no single sample could be analyzed twice, a negative PMA test result meant that the second sample from the positive lot did not contain infectious virus. Furthermore, the three PCR products sent for Sanger-sequencing were found to be negative for HuNoV GI.

### 3.3. Prevalence of HEV in Blueberries

Of the 150 blueberry samples collected, none tested positive for HEV RNA (Table 5). The prevalence of HEV in Canadian blueberries harvested in Quebec and New Brunswick was thus considered to be between 0.00 and 2.43%, within the range estimated by the statistical plan. The Mengo virus was recovered from each method and each sample, with an average of 35.6% recovery. As for cranberries, the recovery of the Mengo virus was greater than 1% for all the samples (17.0–54.2) (Table 5).

## 4. Discussion

Viral contamination of cranberries could occur before, during or after harvest. These berries are in close contact with water throughout the year. Water is used for irrigation and to facilitate harvesting, since the berries float. Irrigating the fields also protects them against freezing [10,11]. The water used comes mainly from rainfall accumulation and snowmelt but can also come from nearby streams [10]. After the harvest, cranberries are usually sent to a receiving station where they are washed and, in some cases, disinfected with peracetic acid or chlorine water before processing [36,37]. The degree of disinfection may vary depending on the receiving station; there is no mandatory control of viral contamination on cranberries in Canada [38]. Contamination could also occur after disinfection if food handlers fail to follow good hygiene practices [7]. Even though cranberries do not have a porous surface like strawberries or raspberries, the abundant use of water from various sources during growth puts this matrix at risk for viral contamination before and during harvest [39]. Two outbreaks of HuNoV involving the contamination of cranberries are on record in the USA [40,41]. Despite studies showing the contamination of fresh and frozen berries by HuNoV and HAV, cranberries were never analysed [18,42,43,44,45]. To our knowledge, the only viral surveillance studies to have included cranberries is a study of HuNoV conducted in China in 2016–2017, which found that 0.83% (1/120 samples) of frozen and 4.12% (5/120 samples) of fresh domestic retailed cranberries were contaminated, while no exports, fresh or frozen, were contaminated [46]. The other study would have considered dried cranberries as the cause of a hepatitis A virus outbreak. However, the source of the outbreak could not be confirmed, and the investigation led to the conclusion that blackberries and shrimps may have been the cause [47]. There are no data on the level of viral contamination of RTE Canadian cranberries, even though Canada is the second largest cranberry producing country in the world [8].

Our results suggest that the risk of contamination of RTE Canadian cranberries by HuNoV or HAV is low to non-existent. The low prevalence (below 5%) is consistent with a previous study of the presence of foodborne viruses on fresh and frozen fruit (blackberries, blueberries, raspberries, strawberries, and pomegranate arils) in Canada, in which 0.36% of samples were positive [18]. Our present results indicate a prevalence of between 0 and 2% (1.28%), which is lower than expected. The number of samples chosen for this pilot study was arbitrary but nevertheless led to a plausible result. It is important to note that the cultivation method, irrigation and harvesting practices all influence the prevalence of foodborne viruses on berries and that prevalence could therefore differ from one country to another. The proximity of cranberry fields to cities or inadequate treatment of sewage could also increase the likelihood of contamination of irrigation water or harvest water [48]. Knowledge of, and insistence on, good hygiene practices and the generalization of sewage treatment in Canada no doubt also reduce the risk of viral contamination.

Pretreatment of virus with PMA and Sanger sequencing made it possible to rule out the presence of potentially infectious HuNoV GI particles on cranberries [35]. In this case, the PMA pre-treatment had to be done on a different sample from the same lot number. Sanger sequencing may not have worked if there was a strong degradation of the fragment. Indeed, the length of the sequenced product is equivalent to 329 bp while the fragment detected by RT-qPCR is only 46 bp. Thus, the presence of free RNA could explain the detection of HuNoV GI RNA in RT-qPCR and degradation of the fragment could explain why it was not detected during sequencing. The low number of genome copies per gram detected in samples could be due to the applied sanitation and may also explain why the sequencing did not work [49]. Further studies are needed to investigate the presence of foodborne viruses in cranberry harvest water and to evaluate the efficiency of viral inactivation methods on cranberries, such as peracetic acid.

In the case of Canadian blueberries, HEV type 3 or 4 contaminations could occur through direct contact of the fruit with feces of wild animals (e.g., deer) since some blueberry fields are located near forested areas. Although seroprevalence is low in wildlife in most countries [50,51], the possibility of transmission from wild species to humans needs to be investigated since foodborne transmission of HEV is still poorly understood [16]. Here, we attempted to reveal the potential for transmission of HEV through blueberries, especially since Canada is the world’s third largest producer of blueberries, and the largest exporter of wild blueberries [52].

Our results suggest that HEV contamination of wild blueberries is very rare or even non-existent in Quebec and New-Brunswick, and that the risk of transmission of this virus to humans through ingestion of contaminated blueberries may be considered minimal in these two regions. A sampling plan including more producers, larger growing areas, and other provinces would provide additional information about the safety of blueberries in Canada. Although not investigated in our study, our results are consistent with HEV seroprevalence and viremia reported for the Canadian cervid population [15]. In other countries, HEV has been found in vegetables, fruits, and water [14,53], and a study conducted in Sicily showed that 1.4% of sampled vegetables and 4.3% of water samples were positive for HEV RNA [44]. However, fruit and vegetable cultivation and harvesting techniques, sources of water [48], and the surrounding environment vary considerably from one country to another, and likely affect the results obtained. The use of pig manure as fertilizer also must play a role in HEV type 3 or 4 transmissions since swine are the main hosts of this virus and seropositivity is widespread in pigs in industrialized countries and in Canada [54,55]. Investigations of other products grown close to the ground are needed to provide a better understanding of HEV epizoology and epidemiology in Canada.

The ISO method of detecting foodborne viruses may underestimate the actual amounts of virus present because of the presence of inhibitors that may interfere with the elution of virions from the sample [56]. Indeed, the high variability of recovered Mengo virus may be explained by the presence of inhibitors. Furthermore, the fruit itself and/or the presence of damage at its surface could lead to an increase in inhibitors and induce variability. Another consideration is that viruses on berries are usually soiled by contaminated water, in which case virions may be highly diluted and thus missed by the sampling method. [57]. A negative result does not mean a viral-free sample. Furthermore, when a viral genome is detected by RT-qPCR, positive confirmation by PMA pretreatment should also be required, as is cell culture (excluding HuNoV) or other means to provide information on the infectious status of the virus, which is as important as detection.

In conclusion, the prevalence observed for HuNoV GI is 1.28%, for both HAV and HuNoV GII it is below 5% in RTE cranberries and for HEV it is under 2.43% in blueberries. Overall, the prevalence of foodborne viruses in Canadian berries is very low, making these products safe to eat for consumers. To our knowledge, this is the first study of the potential contamination of Canadian RTE cranberries and wild blueberries with foodborne viruses (HuNoV, HAV and HEV). Additional studies are required to learn more about the potential presence of foodborne viruses on vegetables and other fruits produced in significant quantities in Canada that are grown close to the ground or near potentially contaminated water.

## Figures and Tables

**Table 1:** Primers, probe sequences, quencher and dye used for detection of virus by RT-qPCR.

Target	Sequence	References
**Mengo Virus**		
Forward primer	GCGGGTCCTGCCGAAAGT	[26]
Reverse primer	GAAGTAACATATAGACAGACGCACAC	[26]
Probe	6FAM-ATCACATTACTGGCCGAAGC-MGBNFQ	[26]
**HuNoV GI**		
Forward primer	CGCTGGATGCGNTTCCAT	[27]
Reverse primer	CCTTAGACGCCATCATCATTTAC	[28]
Probe	6FAM-TGGACAGGAGAYCGCRATCT-TAMRA	[28]
**HuNoV GII**		
Forward primer	ATGTTCAGRTGGATGAGRTTCTCWGA	[29]
Reverse primer	TCGACGCCATCTTCATTCACA	[30]
Probe	6FAM-AGCACGTGGGAGGGCGATCG-TAMRA	[29]
**HAV**		
Forward primer	TCACCGCCGTTTGCCTAG	[31]
Reverse primer	GGAGAGCCCTGGAAGAAAG	[31]
Probe	6FAM-CCTGAACCTGCAGGAATTAA-MGBNFQ	[31]
**HEV**		
Forward primer	CGGTGGTTTCTGGGGTGAC	[24]
Reverse primer	AAGGGGTTGGTTGGATGAATATAG	[24]
Probe	6FAM-TGATTCTCAGCCCTTCG-MGBNFQ	[25]

**Table 3:** Summary of HuNoV GI, HuNoV GII and HAV detection in RTE cranberries harvested in Canada.

Total No. of Samples	Virus	No. of Positive Samples	Mengo Virus Recovery (%)	Recovery Standard Deviation (%)
234	HuNoV GI HuNoV GI IHAV	3 (1.28%, 95% CI 0.27–3.70%) 0 (95% CI 0.00–1.56%) 0 (95% CI 0.00–1.56%)	28.7	1.2–86.9

**Table 4:** Summary of HuNoV-positive cranberry sample results *.

Date of Harvest	Viral RNA Detected	1st RT-qPCR			2nd RT-qPCR	
		Undiluted	Diluted	Mengo Virus Recovery (%)	Undiluted	Diluted
5 October 2021 6 October 2021 10 October 2021	HuNoV GI HuNoV GI HuNoV GI	3.6 * (1/2) ** (0/2) ** (0/2) **	(0/2) ** 7.4 * (1/2) ** 5.3 * (1/2) **	35.2 22.3 21.3	(0/3) ** 39.5 *** (1/3) ** 4.8 * (1/3) **	53.6 * (1/3) ** 39.5 *** (1/3) ** (0/3) **

* Values are presented as genome copies/g. ** Represents the number of wells containing a positive sample. *** Both values were combined, and we are representing the average genome copies/g.

**Table 5:** Summary of HEV detection in blueberries harvested in Canada.

Total No. of Samples	No. of HEV-Positive Samples	Mengo Virus Recovery (%)	Recovery Standard Deviation (%)
150	0 (95% CI 0.00–2.43%)	35.6	17.0–54.2

## Data Availability

The data presented in this study are available on request from the corresponding author.
